# Positive Effect of Propolis on Free Radicals in Burn Wounds

**DOI:** 10.1155/2013/356737

**Published:** 2013-05-23

**Authors:** Pawel Olczyk, Pawel Ramos, Katarzyna Komosinska-Vassev, Jerzy Stojko, Barbara Pilawa

**Affiliations:** ^1^Department of Community Pharmacy, Medical University of Silesia in Katowice, 41-200 Sosnowiec, Poland; ^2^Department of Biophysics, Medical University of Silesia in Katowice, 41-200 Sosnowiec, Poland; ^3^Department of Clinical Chemistry and Laboratory Diagnostics, Medical University of Silesia in Katowice, 41-200 Sosnowiec, Poland; ^4^Center of Experimental Medicine, Medical University of Silesia in Katowice, 40-752 Katowice, Poland

## Abstract

Concentration and properties of free radicals in the burn wounds treated with propolis were examined by the use of electron paramagnetic resonance spectroscopy. Magnetic spin-spin interactions and complex free radicals structures in wound beds were studied. The results were compared to those obtained for silver sulphadiazine used as a standard pharmaceutical agent. The changes of free radicals in the matrix of injury with time of exposition on these substances were tested. The aim of this study was to check the hypothesis about the best influence of propolis on the burn wounds healing. It was confirmed that a relatively lower concentration of free radicals exists in the burn wounds treated with propolis. The homogeneously broadened spectra and a complex free radical system characterize the tested tissue samples. The fastening of spin-lattice relaxation processes in the matrix of injury after treatment with propolis and silver sulphadiazine was observed. Practical usefulness of electron paramagnetic resonance spectroscopy in alternative medicine was proved.

## 1. Introduction

Free radicals are formed in burn wounds matrix in thermolysis processes [1]. As the result of high temperatures effects, the chemical bonds are broken in the molecules of skin structures, and as a result, the molecules with unpaired electrons appear [2]. It is expected that different types of free radicals are formed in skin as a reach molecular object. Because of the contents of unpaired electrons, these molecules or molecular fragments are reactive and unstable units. They may cause major biochemical reactions in skin and in the neighboring tissues [3]. Free radicals interact not only with other radical species but also with diamagnetic molecules. The product may be paramagnetic or diamagnetic [4]. So, it can be seen that oxidative reactions modify chemical structures in a living organism and may cause toxic effects [5]. The influence of free radicals may be long distance, because of chain reactions. The formation of free radicals in skin and in tissues is strongly negative in results. During skin burning free radicals are formed, but proper drugs should still be chosen [6]. Despite therapeutic activity of the drug, it should also quench radical activity. Therefore European propolis (including Polish origin epitherapeutic agent), a natural plant resin that is produced by honey bees, seems to be an optimal agent for the treatment of thermal injuries due to its antimicrobial, anti-inflammatory, antitumor, immunomodulatory, and last but not least antioxidant activity [7–10]. Beneficial biological effect of propolis has been widely used in dermatology for injuries healing, thermal damage and external ulcers therapy, healing time reduction, wound contraction increase, and tissue repair acceleration [11]. The main active compounds responsible for the biological activity of poplar (European) propolis type, including propolis from Polish origin are flavonoids (chrysine, galangine, pinocembrine, and pinobanxine), phenolic acids (caffeic acid, p-cumaric acid, and ferulic acid), and their esters (phenylethyl and 1.1-dimethylallyl) [12, 13]. 

In contrary to the last mentioned natural agent, silver sulphadiazine conventionally used for topical burn therapy, effective in controlling the infection of damaged skin, may lead to prolongation of the wound reepithelization process [10, 14–16].

In this work the effect of propolis on burnt skin repair with increasing time of its action is studied. The changes in free radicals in wound matrix after treatment with propolis, as a natural drug, were compared with those obtained for the standard agent—silver sulphadiazine. Our studies are innovatory, as they show that free radicals in the matrix of burn wounds treated with propolis were marginally expressed. Such studies had not been performed so far probably because of technical and analytical difficulties. 

In our work we proposed electron paramagnetic resonance (EPR) spectroscopy as an experimental tool for the examination of free radicals in burn wounds matrix. This method is based on microwave absorption by the samples located in magnetic field. A new numerical procedure of the spectral analysis is proposed. The performed studies of the effect of propolis on free radicals in burn wounds are a fine example which brings to light their broader applications. Usefulness of the proposed spectroscopic analysis and prepared numerical procedures in alternative medicine was discussed. 

## 2. Material and Methods

### 2.1. Therapeutic Agents

3% propolis ointment, attested by the National Institute of Hygiene (HZ/06107/00) and 1% silver sulfadiazine cream (Lek, Poland) were used.

### 2.2. Tissue Materials

The experimental trial was accepted by the Ethics Committee of the Medical University of Silesia, Poland. Four 16-week-old domestic pigs were implemented for the evaluation of wound repair because of the many similarities between porcine skin and human skin. 72 contact thermal injuries were inflicted according to the methods of Hoekstra et al. [17] and Brans et al. [18]. Experimental animals were housed according to the Good Laboratory Practice Standards. Animals were included into control (*n* = 2) and experimental (*n* = 2) groups. In the control group wounds were treated with physiologic salt (NaCl) to observe the healing process occurring without management (one animal) or with a propolis vehicle in order to exclude its possible effect on the propolis properties (another animal), twice a day, throughout 21 days. In the experimental group, burns were treated with propolis (one animal) or silver sulfadiazine (another animal), twice a day, for three weeks. Biopsies, in three replications, were taken from healthy skin at day 0 and from the wound bed on postburn days 3, 5, 10, 15, and 21. After burn infliction, thermally damaged tissues were rinsed with an antiseptic agent and then treated with propolis, silver sulfadiazine, propolis vehicle, and NaCl, respectively. In the case of burn wounds treated with the propolis, silver sulfadiazine, and propolis vehicle, the wound surface was covered with 0.50–0.75 cm layer of topically applied experimental agent. The wounds were then covered with a woven cotton material. The wounds left by the biopsy were covered with collagen dressing. 

### 2.3. Sample Preparation to EPR Measurements

The tissue samples were placed in the thin walled glass tubes with the external diameter of 3 mm. The mass of the used skin samples was measured. The EPR signals were not measured for the empty glass tubes up to the applied receiver gains (10^5^) and microwave powers (2.2–70 mW). 

### 2.4. EPR Measurements

#### 2.4.1. Conditions of EPR Measurements

Free radicals in the tissue samples were examined by the use of electron paramagnetic resonance spectroscopy. Paramagnetic properties and free radical concentrations in the tissue samples were tested. EPR spectra were measured by the use of an X-band (9.3 GHz) electron paramagnetic resonance spectrometer with magnetic modulation of 100 kHz produced by Radiopan Firm (Poznan, Poland). 

Free radical concentrations were measured at low microwave power of 2.2 mW (attenuation of 15 dB) to avoid microwave saturation effect. The parameters of the EPR spectra of the individual samples recorded with the microwave power of 2.2 mW were compared. Additionally, in order to obtain information about properties of free radicals in tissue samples, the influence of microwave power in the range of 2.2–70 mW on their EPR lines was checked. For the burn wounds treated with propolis and silver sulphadiazine, the lineshape and the parameters of the EPR spectra were compared. 

#### 2.4.2. The Analysed Parameters of the EPR Spectra

The following parameters of EPR spectra of the studied tissue samples were analysed: *g*-factors [±0.0002], amplitudes (*A*) [±0.1], integral intensities (*I*) [±0.2], and linewidths (Δ*B*_pp_)  [±0.02]. Above the maximal errors for amplitude, integral intensities, and linewidths are shown. The error for the *g* values was calculated by the total derivative method. The amplitude and integral intensity rise with increasing concentration of free radicals in the samples. The linewidth depends on paramagnetic properties of the samples.

*g* values were calculated from EPR spectra according to the resonance formula [19]: *g* = *hν*/*μ*_*B*_ *B*_*r*_, where *h* is Planck constant, *ν* is microwave frequency, *μ*_B_ is Bohr magneton, and *B*_*r*_ is resonance magnetic field. Microwave frequency (*ν*) was directly measured by MCM101 recorder produced by EPRAD Firm (Poznan, Poland). The *B*_*r*_ values were determined from the EPR spectra.

The influence of microwave power on the EPR spectra (in order to obtain information about spin-lattice relaxation processes and the type of line broadening) was tested. Changes of amplitudes (*A*), integral intensities (*I*), and linewidths (Δ*B*_pp_) with microwave power in the range 0.7–70 mW were evaluated. Spin-lattice relaxation processes in the samples depend on microwave saturation of the EPR lines. The microwave power of saturation of EPR lines increases for the faster spin-lattice relaxation processes in the sample. Homogeneous or inhomogeneous broadening of EPR lines differ in correlations between the parameters of the EPR spectra and the microwave power [19].

#### 2.4.3. The Analysed Lineshape Parameters of EPR Spectra

**Figure 1:**
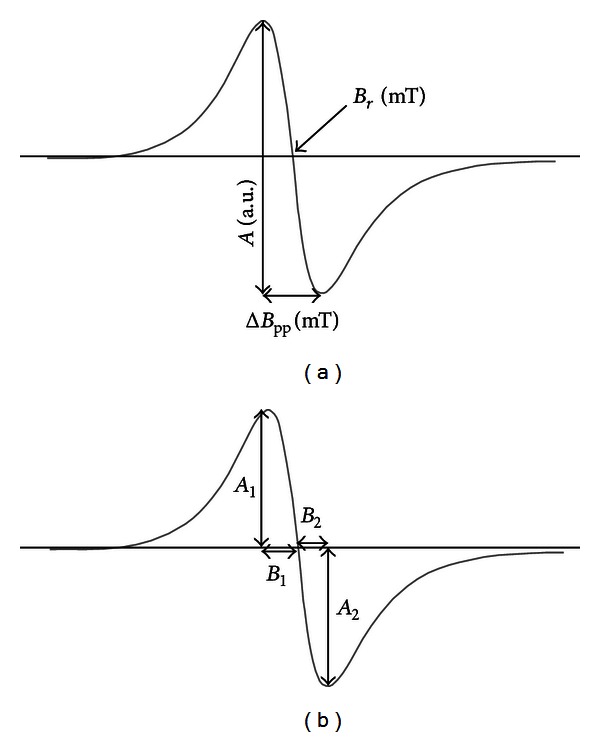
The first derivative EPR spectrum with the parameters: amplitude (*A*) [±0.1], linewidth (Δ*B*_pp_) [±0.02], and resonance magnetic field (*B*_*r*_) [±0.01] (a). The analysed lineshape parameters of the EPR spectra: *A*_1_, *A*_2_, *B*_1_, and *B*_2_ (b). The values of *A*_1_/*A*_2_ [±0.02] and *B*_1_/*B*_2_ [±0.02] were calculated.

The effect of microwave power on the lineshape of the EPR spectra was examined to check the hypothesis about complex structure of free radicals in the samples. The theory of electron paramagnetic resonance indicates that the shape of EPR spectra changes with the microwave power when several different groups of free radicals exist in the sample [19]. The lineshape parameters *A*_1_/*A*_2_ and *B*_1_/*B*_2_ of the EPR spectra were analysed. The values of *A*_1_, *A*_2_, *B*_1_, and *B*_2_ are presented in Figure 1. The lineshape parameters *A*_1_/*A*_2_ and *B*_1_/*B*_2_ were determined for the EPR spectra measured in the range of microwave power from 2.2 mW to 70 mW. The correlations between the *A*_1_/*A*_2_ and *B*_1_/*B*_2_ parameters and microwave power were analysed.

#### 2.4.4. Determination of Free Radicals Concentration in Tissue Samples

Concentrations of free radicals (*N*) in wounds treated with propolis and silver sulphadiazine were compared. The free radicals concentration (*N*) was determined as a value proportional to the integral intensity (*I*) of EPR spectra according to the formula [19, 20]:

(1) $$\begin{array}{c} N=\frac{N_{u}\left[\left(W_{u}A_{u}\right)/I_{u}\right]}{\left[I/WAm\right]\;}, \end{array}$$

where *N*_*u*_ is the number of paramagnetic center (1.2 × 10^19^ spin) in the ultramarine reference, *W* and *W*_*u*_ are the receiver gains for the sample and the ultramarine, *A* and *A*_*u*_ are the amplitudes of ruby signal for the sample and the ultramarine, *I* and *I*_*u*_ are the integral intensities for the sample and ultramarine,  and *m* is the mass of the sample.

The values of integral intensities were numerically calculated by double integration of the first-derivative EPR spectra. Ultramarine was used as the reference for free radical concentrations. The integral intensities of the EPR spectra of tested samples were compared to the integral intensity of the ultramarine spectrum. A ruby crystal (Al_2_O_3_ : Cr^3+^) permanently placed in a resonance cavity was used as the second reference. For each sample and for the ultramarine, the EPR line of a ruby crystal was detected with the same receiver gain and at the same microwave power. By the use of two references (ultramarine and a ruby crystal) the low maximal error for determined values of free radical concentrations in the samples *N* [±0.2 × 10^22^ spin/g] was obtained.

## 3. Results

**Figure 2:**
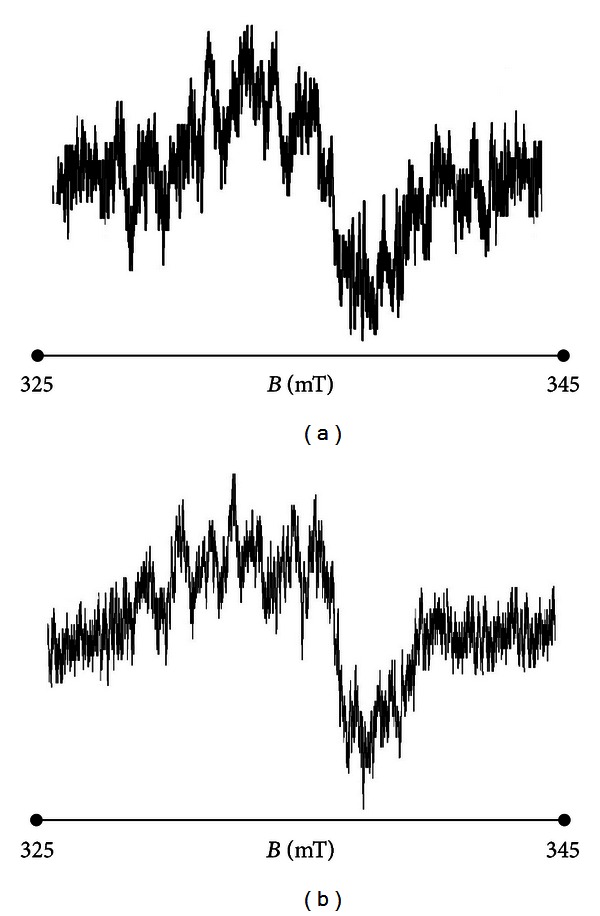
EPR spectra of burn wounds matrix treated with propolis and silver sulphadiazine after 3 days of the therapy. The spectra were measured with the use of a microwave power of 2.2 mW at room temperature.

The performed spectroscopic studies indicated that free radicals exist in all tested tissue samples. The EPR spectra were obtained for all samples. Broad asymmetrical EPR lines were measured for burn wounds treated with propolis and silver sulphadiazine. The resonance lines were also measured for wounds treated with physiological salt and the propolis vehicle. The shape of the detected EPR spectra of samples isolated from wounds treated with tested substances was similar. The exemplary EPR spectra of samples extracted from burn wounds treated with propolis and silver sulphadiazine after 3 days are shown in Figure 2. 

The EPR parameters of the studied tissue samples depend on the type of drug and the time of its influence on the wound. Linewidths (Δ*B*_pp_), amplitudes (*A*), integral intensity (*I*), and free radical concentrations (*N*) in burn wounds matrix treated with physiological salt, propolis vehicle, propolis, and silver sulphadiazine are presented in Tables 1, 2, 3, and 4, respectively. 

The lowest values of amplitude (*A*) and integral intensity (*I*) of EPR lines of tissue samples were measured for burn wounds treated with propolis after 21 days of the therapy (Tables 1–4). The lowest value of the linewidth (Δ*B*_pp_) of EPR spectra was also observed for burn wounds treated with propolis after 21 days (Tables 1–4). The changes of the parameters of the EPR spectra (*A*, *I*, and  Δ*B*_pp_) with increasing time of interactions with the physiological salt, propolis vehicle, propolis, and silver sulphadiazine are not regular (Tables 1–4) because of the complex, multicomponent free radical system in the samples. Free radical concentration (*N*) in all samples changes with the time of the pharmacotherapy (Tables 1–4). The lowest free radical concentrations characterize burn wounds treated with propolis after 21 days of the therapy (Table 3). The free radical concentration (*N*) in burn wounds at the end of the propolis therapy (21 days) was lower (Table 3) than in the matrix of injury treated with physiological salt (Table 1). It was pointed out that propolis strongly quenches free radicals. Such an effect was not observed for the standard drug, silver sulphadiazine. A considerably higher free radical formation was observed in burn wounds treated with silver sulphadiazine after 21 days of its usage (Table 4). The *g*values of the EPR lines of tested samples were near 2.00. It is a typical value for free radicals with unpaired electrons located on oxygen and carbon atoms. The location of unpaired electrons on different atoms (O, C) and in major structures indicates a complex character of free radical system in skin. A complex character of the free radical system in samples was proved by the measurements of the asymmetry parameters of the EPR spectra recorded at different microwave powers. The lineshape of the EPR spectra of all tested samples changes with microwave power. The influence of microwave power (*M*) on the lineshape parameters *A*_1_/*A*_2_ and *B*_1_/*B*_2_ of the EPR spectra is presented in Tables 5, 6, 7, and 8, respectively. The ranges of the values of the parameters *A*_1_/*A*_2_ and *B*_1_/*B*_2_ of the EPR spectra for all samples can be seen in Tables 5–6. The lowest and the highest values of the parameters *A*_1_/*A*_2_ and *B*_1_/*B*_2_ are marked by bold letters. Strong changes of these parameters with microwave power indicated that several groups of free radicals exist in tested samples.

**Figure 3:**
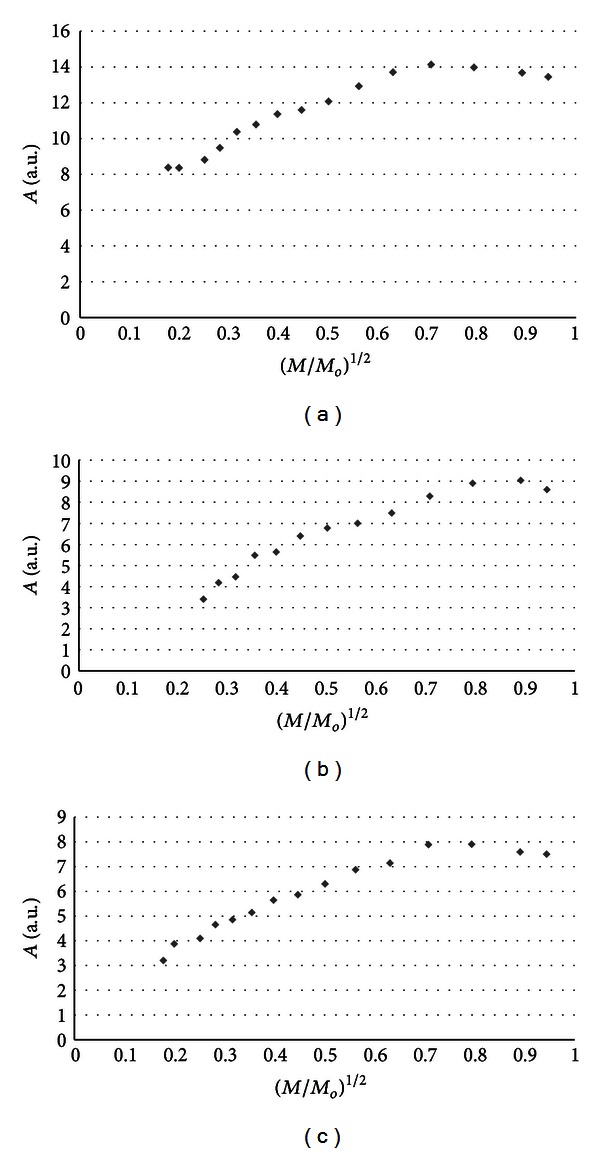
The influence of the microwave power (*M*) on the amplitude (*A*) [±0.1] of EPR spectra of the burn wounds after treatment with physiological salt for 0 (a), 5th (b), and 21st (c) day. *M*_*o*_ (70 mW) is the total microwave power produced by klystron. The spectra were measured at room temperature.

**Figure 4:**
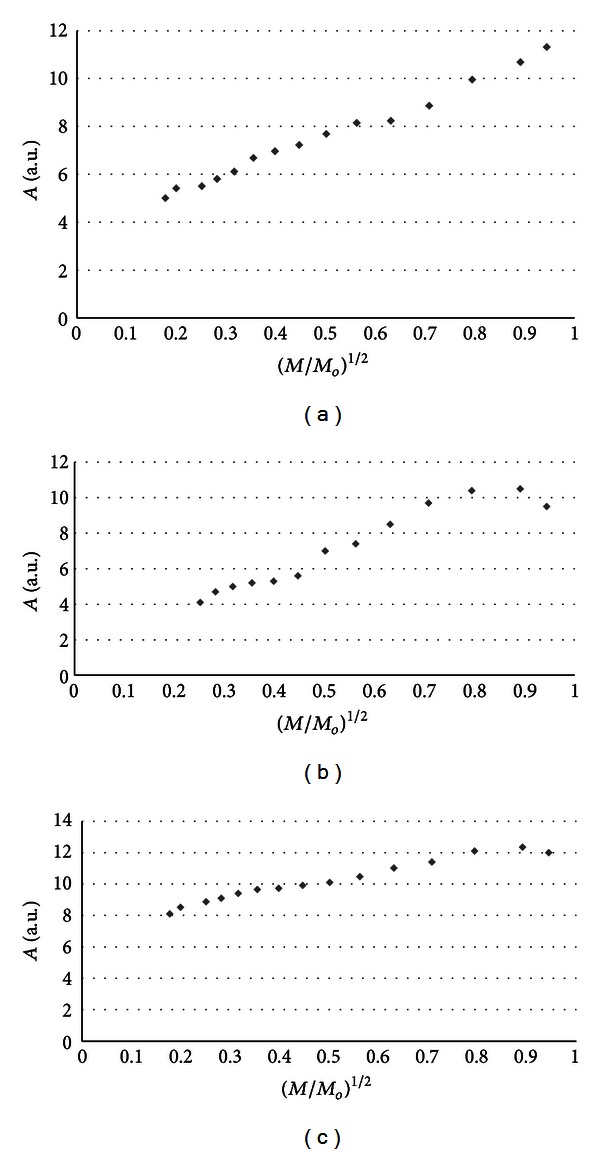
The influence of the microwave power (*M*) on the amplitude (*A*) [±0.1] of EPR spectra of the burn wounds after treatment with propolis vehicle for 0 (a), 5th (b), and 21st (c) day. *M*_*o*_ (70 mW) is the total microwave power produced by klystron. The spectra were measured at room temperature.

**Figure 5:**
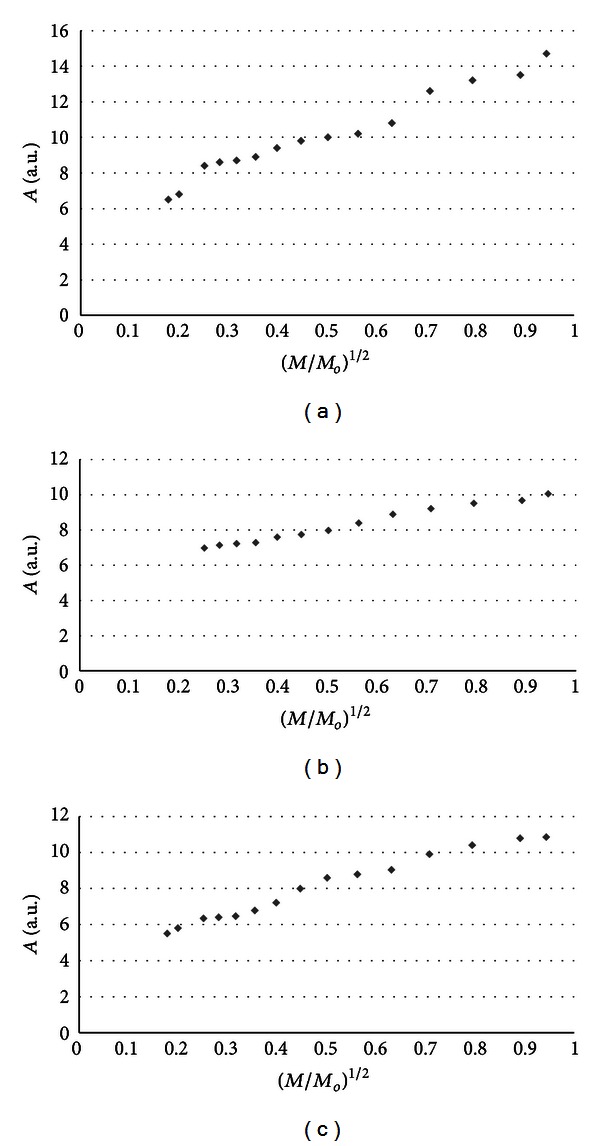
The influence of the microwave power (*M*) on the amplitude (*A*) [±0.1] of EPR spectra of the burn wounds after treatment with propolis for 0 (a), 5th (b), and 21st (c) day. *M*_*o*_ (70 mW) is the total microwave power produced by klystron. The spectra were measured at room temperature.

**Figure 6:**
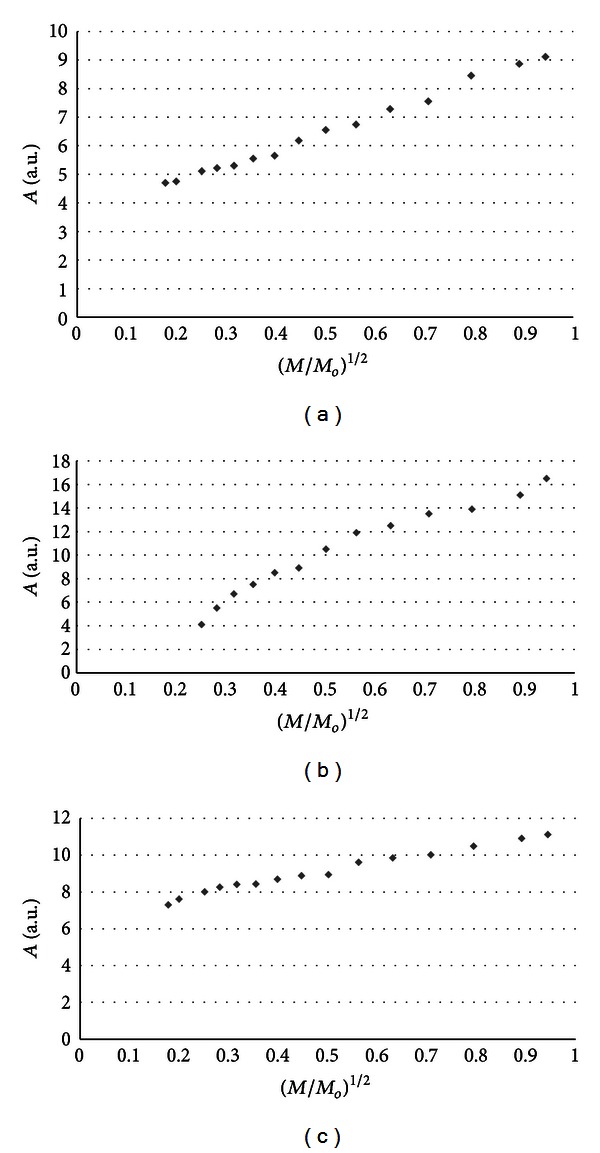
The influence of the microwave power (*M*) on the amplitude (*A*) [±0.1] of EPR spectra of the burn wounds after treatment with sulphadiazine for 0 (a), 5th (b), and 21st (c) day. *M*_*o*_ (70 mW) is the total microwave power produced by klystron. The spectra were measured at room temperature.

**Figure 7:**
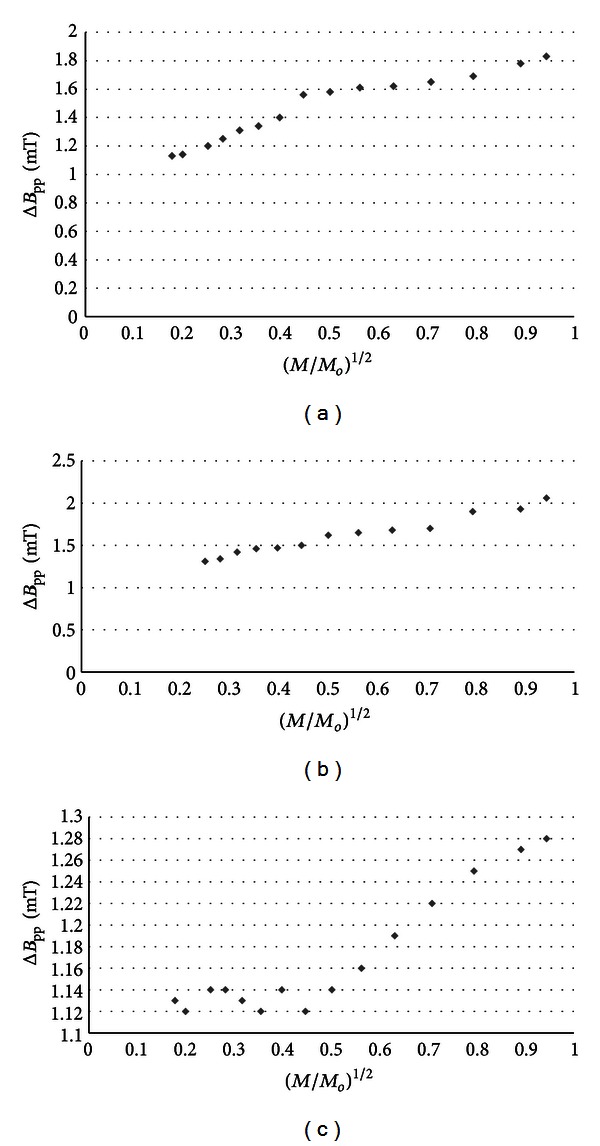
The influence of the microwave power (*M*) on linewidth (Δ*B*_pp_) [±0.02] of EPR spectra of the burn wounds after treatment with physiological salt for 0 (a), 5th (b), and 21st (c) day. *M*_*o*_ (70 mW) is the total microwave power produced by klystron. The spectra were measured at room temperature.

**Figure 8:**
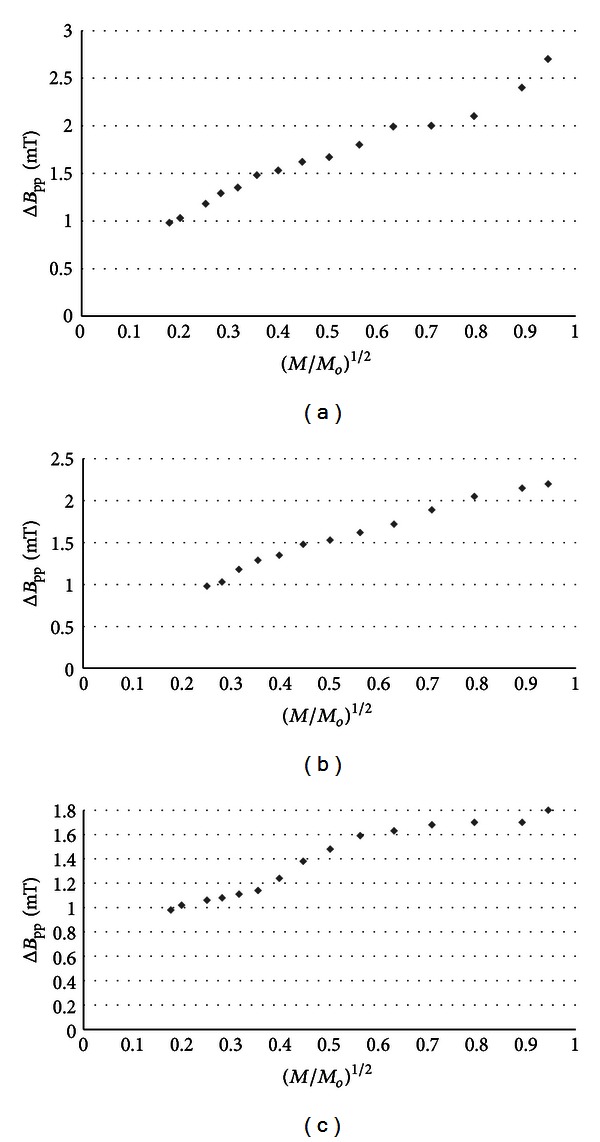
The influence of the microwave power (*M*) on linewidth (Δ*B*_pp_) [±0.02] of EPR spectra of the burn wounds after treatment with propolis vehicle for 0 (a), 5th (b), and 21st (c) day. *M*_*o*_ (70 mW) is the total microwave power produced by klystron. The spectra were measured at room temperature.

**Figure 9:**
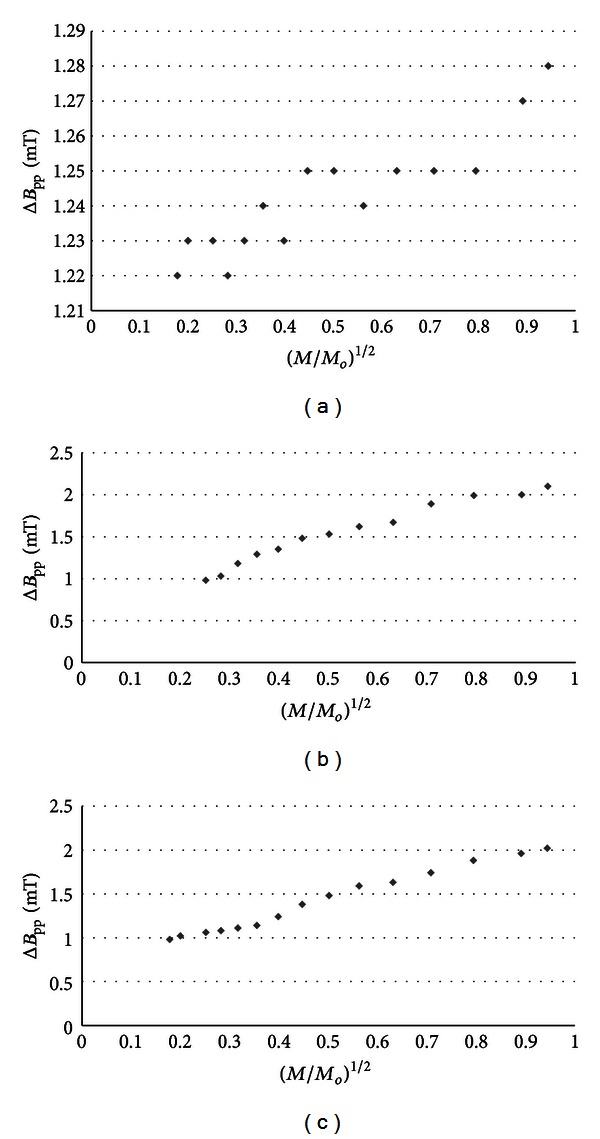
The influence of the microwave power (*M*) on linewidth (Δ*B*_pp_) [±0.02] of EPR spectra of the burn wounds after treatment with propolis for 0 (a), 5th (b), and 21st (c) day. *M*_*o*_ (70 mW) is the total microwave power produced by klystron. The spectra were measured at room temperature.

**Figure 10:**
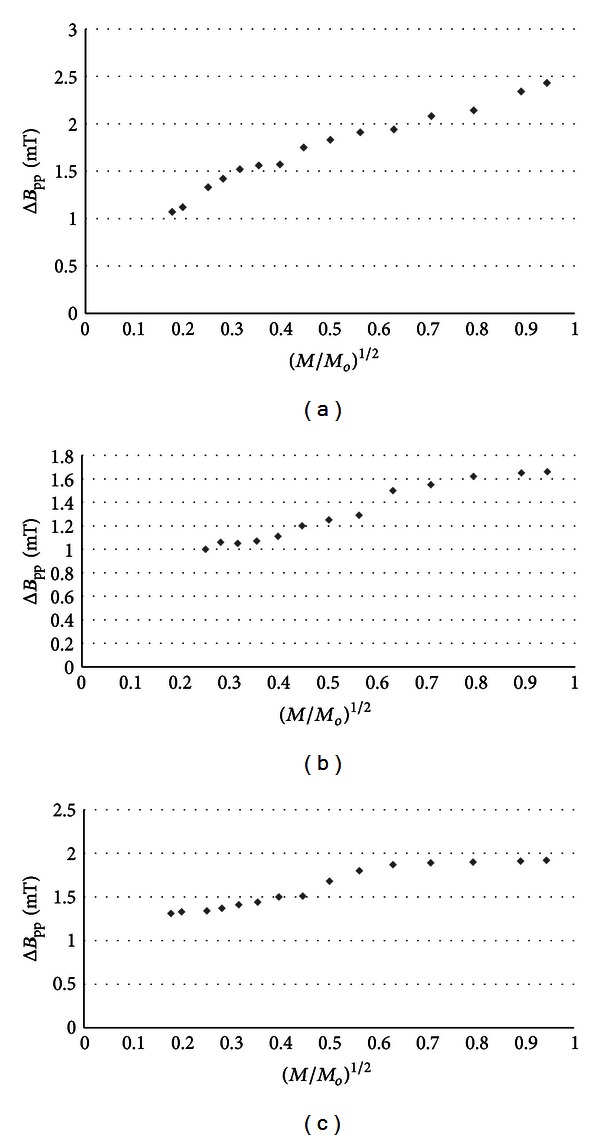
The influence of the microwave power (*M*) on linewidth (Δ*B*_pp_) [±0.02] of EPR spectra of burn wounds after treatment with silver sulphadiazine for 0 (a), 5th (b), and 21st (c) day. *M*_*o*_ (70 mW) is the total microwave power produced by klystron. The spectra were measured at room temperature.

The influence of microwave power on amplitudes (*A*) of burn wounds after treatment with physiological salt (Figure 3), propolis vehicle (Figure 4), propolis (Figure 5), and silver sulphadiazine (Figure 6) was obtained. The changes of linewidths (Δ*B*_pp_) of the EPR spectra of burn wounds after treatment with physiological salt, propolis vehicle, propolis, and silver sulphadiazine are shown in Figures 7, 8, 9, and 10, respectively. Amplitudes of all recorded EPR spectra rise with increasing microwave power (Figures 3–6). A low increase of linewidths (Δ*B*_pp_) of the EPR spectra was observed (Figures 7–10). The correlations presented in Figures 3–10 were characteristics for free radicals homogeneously distributed in the samples.

Amplitudes of all recorded EPR spectra rise with increasing microwave power (Figures 3–6). Amplitudes of the EPR lines decrease with higher values of microwave power for burn wounds treated with physiological salt (Figure 3), propolis vehicle after 5 days (Figure 4(b)), and after 21 days (Figure 4(c)). A decrease of amplitudes of the EPR lines of injury matrix treated with propolis (Figure 5) and silver sulphadiazine (Figure 6) at higher microwave powers was not observed. The absence of microwave saturation of the EPR spectra (Figures 5 and 6) indicated that relatively faster spin-lattice relaxation processes exist in the wounds treated with propolis rather than in the those treated with physiological salt and propolis vehicle (Figures 3 and 4). An increase of linewidths (Δ*B*_pp_) of the EPR spectra was observed (Figures 7–10). The broadening of EPR lines with increasing microwave power is observed only for free radicals homogeneously distributed in the samples.

## 4. Discussion

The application of the X-band electron paramagnetic resonance spectroscopy for examination of burn wounds pointed out that free radicals with unpaired electrons exist in all samples. For all wound samples after treatment with both physiological salts or propolis vehicle and propolis or silver sulphadiazine, EPR spectra were detected (Figure 2). The parameters of the spectra depend on the substance interacting with the wound matrix. Free radicals with *g* values of 2.00 are responsible for the EPR lines. The EPR spectra did not diminish in time after the treatment, so it can be stated that free radicals in tested samples are stable.

The interactions of free radicals in skin and in neighboring tissues may be responsible for a lot of toxic effects and modifications of their structures. It is a negative effect which may destroy normal functions of biological structures in the organism [6]. Free radicals take part in biochemical reactions in the healthy organism, but they are the agents which initiate damages in the organism, since a lot of illnesses are accompanied by a high production of freer radicals in the living organism [3]. The well-known example of destructive influence of free radicals on tissues is the lipid peroxidation process which is initiated by free radicals and accompanied by free radical transformation [6]. The products of lipid peroxidation are diamagnetic and are not reactive, but they are the components of modified biological structures; therefore, their functions may be destroyed [5]. Therapeutic methods should be based on the pharmaceuticals which do not produce free radicals. The best substances are those which quench free radicals in skin and tissues. 

In our work it was found that the substance quenching free radicals in skin is propolis. The optimal method of propolis treatment of the skin of burnt wounds was found. The effect of quenching free radicals in burn wounds was the highest after 21 days of the therapy (Table 3).

The effect of propolis on free radicals in skin is the basis of safety of its application in the therapy of burn wounds. This important problem of alternative medicine, which we are interested in, had not been tested and discussed so far. It is probably caused by the difficulties in chemical analysis of free radicals in skin. In our work we proposed a physical method such as electron paramagnetic resonance spectroscopy to confirm the usefulness of the exemplary drug such as propolis and to determine the time of skin therapy. The usefulness of propolis as a free radical quencher was confirmed, and moreover, it was found that 21 days of its effect on skin are the best time of therapy. 

Contrary to chemical methods, EPR spectroscopy is not destructive for tested samples. The agents, interacting on the samples during the EPR studies, are magnetic field and microwaves. During electron paramagnetic resonance measurements of free radicals, relatively low magnetic fields with magnetic induction B about 331–335 mT are used. Microwaves with frequency of 9.3 GHz are not destructive for the samples. The EPR tests are safe for the molecular structures of the samples and for the persons who led the examination. EPR measurements need only a small number of the samples which are located in the glass tubes in the resonance cavity of the spectrometer. EPR spectroscopic tests give important information about paramagnetic properties of the skin samples and about free radical concentration in them [19, 20].

Stable free radicals with concentrations in the range 10^22^–10^23^ spin/g were found in examined tissue samples (Tables 1–4). Free radical concentrations in wounds after treatment with propolis during 21 days (8.9 × 10^22^ spin/g, Table 3) were one order lower (10^22^ spin/g) than in the skin treated with physiological salt (10^23^ spin/g, Table 1) and propolis vehicle (10^23^ spin/g, Table 2). The effect of quenching free radicals with propolis is clearly visible. The silver sulphadiazine, which is conventionally applied in the therapy of burns [14, 15] produces free radicals in skin (Table 4). The free radical concentrations in burn wounds after treatment with silver sulphadiazine was 31.8 × 10^22^ spin/g (Table 4), while its value for wounds treated with physiological salt was 13.7 × 10^22^ spin/g (Table 1). The determined values of free radicals concentrations in the matrix of burn wounds (Tables 1–4) indicate that propolis is the best substance to use in the tested medicinal example.

The amplitudes, integral intensities (*I*), and linewidths (Tables 1–4) of the EPR spectra of the tested samples are different depending on the interacting substances. The amplitudes and integral intensities affect the values of free radical concentrations which are discussed previously. The broad EPR lines with high values of linewidths (Tables 1–4) indicate that the distances between free radicals in tissue samples are low and that there exist strong dipolar interactions in their structures.

Besides free radicals concentrations, EPR results show the properties of the free radicals in the studied samples. Free radicals in all tested samples have *g* values near 2.00, so it is expected that oxygen and carbon free radicals mainly exist in the skin. The changes of the lineshape of the EPR spectra and the tested lineshape parameter *A*_1_/*A*_2_ and *B*_1_/*B*_2_ (Tables 5–8) with increasing microwave power proved the existence of different groups of free radicals in burn wounds independently of the applied substances. Such changes of the lineshape parameters are not observed in samples with only one group of free radicals. The studies of the complex character of the free radical system in tissue samples will be developed in our next EPR studies.

Free radicals are homogeneously distributed in all tested samples. It is confirmed by the character of changes of amplitudes (Figures 4–6) and linewidths (Figures 7–10) with increasing microwave power. The amplitudes of the examined EPR lines rise with increasing microwave power, reach the maximum, and then they begin to decrease in the case of wounds treated with physiological salt (Figure 3) and propolis vehicle (Figure 4). The amplitudes of the EPR lines of burns treated with propolis (Figure 5) and silver sulphadiazine (Figure 6) increase with the microwave power in the whole used range. However, the saturation effect was not observed. The linewidths of the EPR spectra increase with the increase of microwave power for all tested samples (Figures 7–10). These correlations (Figures 3–10) are typical for free radicals homogeneously distributed in the samples [19]. Homogeneous distribution of free radicals in the samples (Figures 3–10) indicates high quality of experiments of wound treatment with tested substances. The properly performed pharmacotherapy with homogeneous interactions of the substances in the whole volume of the skins was spectroscopically confirmed. The continuous microwave saturation of EPR spectra, which was used in this work in order to examine the homogeneous distribution of free radicals in wounds, may be proposed for other application in alternative medicine. 

The saturation of the EPR lines of the tested samples (Figures 3 and 4) pointed out that slow spin-lattice relaxation processes exist in wounds treated with physiological salt and propolis vehicle. The absence of the saturation effect in the EPR spectra of burns treated with propolis and silver sulphadiazine (Figures 5 and 6) indicates relatively faster spin-lattice relaxation processes in these samples. Broad EPR spectra of the analysed tissue samples together with high values of linewidths (Tables 1–4) are characteristic for strong dipolar interactions between free radicals in the samples [19]. Dipolar interactions increase with decreasing distances between unpaired electrons of free radicals [19, 20]. Therefore, short distances between free radicals in samples were brought to light by EPR lines. High free radical concentrations (Tables 1–4) in the tested samples show a probability of their strong interactions with paramagnetic oxygen molecules [19]. 

It was concluded that the complex free radical system characterizes burn wounds for both propolis and silver sulphadiazine. Oxygen and carbon free radicals mainly exist in the tested samples. Continuous microwave saturation of EPR spectra pointed out that free radicals are homogeneously distributed in the tested samples. Strong dipolar interactions exist in the examined samples. Relatively faster spin-lattice relaxation processes exist in wounds after treatment with both propolis and silver sulphadiazine rather than in the injuries treated with physiological salt and propolis vehicle.

The performed studies confirmed the usefulness of electron paramagnetic resonance spectroscopy and numerical analysis of EPR spectra in the examination of the influence of propolis and other pharmacological substances on the burn wounds. The free radical tests were proposed to practical application in alternative medicine. The best action of propolis on free radicals in skin was proved. 

The beneficial antioxidant activity of propolis is of utmost importance, since the excess of free radicals expressed during oxidative stress, as a consequence of prooxidant-antioxidant imbalance, results in the nonhealing state [21]. Moreover, the free radicals load persisting over a long period of time in healing wound bed leads to the continuous tissue damage and prolonged inflammatory state. A considerably lower free radical concentration in the wound matrix treated with propolis than in the skin treated with silver sulphadiazine corresponds with results of Berretta et al. [22] describing the propolis potential in the tissue repair stimulation. The increased healing action attributed to propolis antioxidative properties may be connected with the inhibition of lipid peroxidation, attenuation of the reperfusion consequences, the prevention of the matrix metalloproteinase overexpression, and endothelial injury [22–25].

We propose the EPR method as an additional method for the medical and pharmaceutical studies. EPR is a useful method of testing of free radicals in cells and tissues [26–29], melanin biopolymers [30], and drugs [31–33]. The EPR examination of free radicals contents in different types of tumor cells [26, 27] and tumor cells under photodynamic therapy [27] is known. Free radicals appear in natural evolution of skin tumor cells; they are formed by laser irradiation in tumor cells with accumulated photosensitizers. Laser irradiation causes excitation of photosensitizer, and when it comes to the ground state, the electromagnetic waves form free radicals in tumor cells and singlet oxygen. The best conditions of laser irradiation during photodynamic therapy were tested by EPR [27]. EPR studies of photodynamic processes are especially important for dermatology as well as the EPR studies of melanin biopolymers [29] and their complexes with drugs [30]. Melanins exist in skin, and EPR characterization of these polymers may be applied in clinical practice. Melanin free radicals take part in binding drugs to these polymers [29, 30]. The activity of the o-semiquinone free radicals of melanin in the reactions with drugs may be analysed by comparative EPR tests of the original melanin and melanin complexes with individual drugs [30]. Next example of clinical application of EPR method is studies of antioxidative properties of bee pollen extracts by the use of DPPH as a paramagnetic probe [31]. EPR was applied to the analysis of free radicals formation in gamma [32] and thermally sterilized drugs [33]. The best conditions of drug sterilization were spectroscopically searched. The sterilized drugs, especially the drugs interacting with skin, should not contain high amounts of free radicals. Free radical concentrations in irradiated and thermally treated drugs were examined by EPR spectroscopy [33]. In our work we presented a fine example of EPR application in alternative medicine. Free radicals in burn wound treated by propolis were broadly characterized. Both the clinical and pharmaceuticals aspects of EPR analysis were brought to light.

## 5. Conclusions

Electron paramagnetic resonance studies of burn wounds pointed out that free radicals exist in all tested samples independently of the type of the pharmacological factor. However, a strong positive effect of propolis on free radical contents in wounds was proved. The free radical concentration in wounds treated with propolis was considerably lower than in injuries treated with the standard drug, silver sulphadiazine. The usefulness of EPR spectroscopy for the examination of the drug influence on the wound matrix was proved.

## Figures and Tables

**Table 1:** Free radical concentration (*N*) in burn wound matrix after treatment with physiological salt and the EPR spectra parameters: amplitude (*A*), integral intensity (*I*), and linewidth (Δ*B*_pp_). The data for the measurement were taken with the use of a microwave of 2.2 mW at room temperature.

Day of treatment	Δ*B*_pp_ [±0.02] (mT)	*A* [±0.1] (a.u.)	*I* [±0.2] (a.u.)	*N* × 10^22^ [±0.2 × 10^22^] (spin/g)
0	1.13	6.6	8.4	2.3
3	1.27	6.4	10.3	28.7
5	1.31	3.2	5.2	14.5
10	1.24	4.8	7.3	20.3
15	1.24	1.9	3.0	8.4
21	1.01	4.7	4.9	13.7

**Table 2:** Free radical concentration (*N*) in burn wound matrix after treatment with propolis vehicle and the EPR spectra parameters: amplitude (*A*), integral intensity (*I*), and linewidth (Δ*B*_pp_). The data for the measurement were taken with the use of a microwave of 2.2 mW at room temperature.

Day of therapy	Δ*B*_pp_ [±0.02] (mT)	*A* [±0.1] (a.u.)	*I* [±0.2] (a.u.)	*N* × 10^22^ [±0.2 × 10^22^] (spin/g)
0	1.33	4.5	8.0	22.3
3	1.17	0.9	1.2	3.3
5	0.99	4.1	4.0	11.2
10	1.18	7.0	9.7	27.1
15	1.44	5.1	10.6	29.5
21	1.51	6.7	15.4	42.9

**Table 3:** Free radical concentration (*N*) in burn wound matrix after treatment with propolis and the EPR spectra parameters: amplitude (*A*), integral intensity (*I*), and linewidth (Δ*B*_pp_). The data for the measurement were taken with the use of a microwave of 2.2 mW at room temperature.

Day of therapy	Δ*B*_pp_ [±0.02] (mT)	*A* [±0.1] (a.u.)	*I* [±0.2] (a.u.)	*N* × 10^22^ [±0.2 × 10^22^] (spin/g)
0	1.22	4.6	6.9	19.3
3	0.95	7.2	6.5	18.1
5	0.98	5.1	5.0	13.9
10	0.88	6.4	4.8	13.4
15	0.77	5.9	3.4	9.4
21	0.98	3.5	3.2	8.9

**Table 4:** Free radical concentration (*N*) in burn wound matrix after treatment with silver sulphadiazine and the EPR spectra parameters: amplitude (*A*), integral intensity (*I*), and linewidth (Δ*B*_pp_). The data for the measurement were taken with the use of a microwave of 2.2 mW at room temperature.

Day of therapy	Δ*B*_pp_ [±0.02] (mT)	*A* [±0.1] (a.u.)	*I* [±0.2] (a.u.)	*N* × 10^22^ [±0.2 × 10^22^] (spin/g)
0	1.07	2.9	3.4	9.5
3	0.95	4.7	4.3	12.0
5	1.00	8.2	8.2	22.9
10	0.75	2.7	1.5	4.2
15	1.23	3.5	5.3	14.8
21	1.31	6.6	11.4	31.8

**Table 5:** The influence of microwave power (*M*) on the lineshape parameters *A*_1_/*A*_2_ and *B*_1_/*B*_2_ of the EPR after treatment with physiological salt.

Day of treatment	0	0	5	5	21	21
*M*/*M*_*o*_	*A*_1_/*A*_1_	*B*_1_/*B*_2_	*A*_1_/*A*_1_	*B*_1_/*B*_2_	*A*_1_/*A*_1_	*B*_1_/*B*_2_
(a.u.)	[±0.02]	[±0.02]	[±0.02]	[±0.02]	[±0.02]	[±0.02]
0.0316	1.31	**2.36**	0.94	**1.50**	**0.43**	1.50
0.0398	**1.54**	2.14	1.17	1.04	**2.75**	**0.20**
0.0631	0.85	1.63	0.89	0.58	1.43	**1.95**
0.0794	1.36	1.39	**0.41**	0.68	1.00	1.87
0.1000	1.32	1.44	1.00	0.90	1.17	0.70
0.1259	1.03	1.18	1.55	0.75	0.75	0.91
0.1585	0.61	2.31	1.27	0.90	1.00	1.14
0.1995	0.94	0.98	1.19	0.43	1.18	1.56
0.2512	**0.51**	0.83	**1.65**	1.04	1.38	1.04
0.3162	0.80	0.78	0.83	0.89	1.36	0.82
0.3982	0.93	0.82	0.98	1.47	0.76	0.77
0.5011	1.22	1.13	0.79	0.95	0.75	0.82
0.6309	1.00	0.83	0.91	0.75	1.16	0.74
0.7944	0.67	0.93	0.77	0.63	0.84	1.59
0.8913	0.94	**0.07**	0.58	**0.38**	1.23	1.32

**Table 6:** The influence of microwave power (*M*) on the lineshape parameters *A*_1_/*A*_2_ and *B*_1_/*B*_2_ of the EPR after treatment with propolis vehicle.

Day of treatment	0	0	5	5	21	21
*M*/*M*_*o*_	*A*_1_/*A*_1_	*B*_1_/*B*_2_	*A*_1_/*A*_1_	*B*_1_/*B*_2_	*A*_1_/*A*_1_	*B*_1_/*B*_2_
(a.u.)	[±0.02]	[±0.02]	[±0.02]	[±0.02]	[±0.02]	[±0.02]
0.0316	1.53	0.59	1.20	1.11	0.65	1.01
0.0398	**1.87**	0.77	0.86	1.41	1.00	0.72
0.0631	0.79	1.86	1.41	1.18	**1.69**	**0.41**
0.0794	0.68	1.09	1.22	**2.14**	0.58	0.94
0.1000	1.18	1.76	0.67	0.71	**0.42**	0.77
0.1259	1.04	**1.88**	0.54	**0.69**	0.93	**2.16**
0.1585	0.84	0.56	0.74	1.06	1.25	1.05
0.1995	1.04	**0.40**	0.65	1.58	0.43	1.06
0.2512	1.17	1.07	1.03	0.78	1.11	0.70
0.3162	0.46	0.61	0.71	1.41	1.11	0.70
0.3982	0.51	2.18	0.73	0.98	0.70	1.75
0.5011	1.67	1.39	**0.45**	1.17	0.46	1.66
0.6309	**0.40**	1.03	1.40	1.09	1.25	1.28
0.7944	1.46	1.84	**1.65**	1.03	0.65	0.97
0.8913	0.57	0.82	0.60	0.79	0.53	0.88

**Table 7:** The influence of microwave power (*M*) on the lineshape parameters *A*_1_/*A*_2_ and *B*_1_/*B*_2_ of the EPR after treatment with propolis.

Day of treatment	0	0	5	5	21	21
*M*/*M*_*o*_	*A*_1_/*A*_1_	*B*_1_/*B*_2_	*A*_1_/*A*_1_	*B*_1_/*B*_2_	*A*_1_/*A*_1_	*B*_1_/*B*_2_
(a.u.)	[±0.02]	[±0.02]	[±0.02]	[±0.02]	[±0.02]	[±0.02]
0.0316	1.25	1.48	0.80	1.05	0.74	1.05
0.0398	1.00	0.72	1.30	1.09	0.96	0.78
0.0631	1.76	1.03	0.77	2.27	**0.42**	**0.66**
0.0794	1.75	2.15	0.94	1.24	1.25	**2.27**
0.1000	**1.83**	**2.45**	1.08	2.53	0.61	1.72
0.1259	1.41	1.00	0.88	0.97	1.40	0.94
0.1585	1.30	0.92	0.95	1.62	0.71	1.92
0.1995	0.92	**0.57**	1.16	**2.46**	**1.74**	1.70
0.2512	1.39	0.88	1.27	**0.47**	0.98	1.79
0.3162	0.74	0.78	**1.42**	0.54	1.00	1.12
0.3982	**0.53**	1.58	0.80	1.21	1.00	1.94
0.5011	0.58	1.09	0.93	1.99	1.04	1.90
0.6309	0.76	0.72	0.97	0.93	1.200	1.55
0.7944	0.58	1.28	0.69	0.85	1.13	1.25
0.8913	0.57	1.11	**0.65**	1.20	0.51	0.79

**Table 8:** The influence of microwave power (*M*) on the lineshape parameters *A*_1_/*A*_2_ and *B*_1_/*B*_2_ of the EPR after treatment with silver sulphadiazine.

Day of treatment	0	0	5	5	21	21
*M*/*M*_*o*_	*A*_1_/*A*_1_	*B*_1_/*B*_2_	*A*_1_/*A*_1_	*B*_1_/*B*_2_	*A*_1_/*A*_1_	*B*_1_/*B*_2_
(a.u.)	[±0.02]	[±0.02]	[±0.02]	[±0.02]	[±0.02]	[±0.02]
0.0316	0.61	1.90	1.69	0.94	**1.57**	**0.74**
0.0398	0.88	1.23	**0.47**	0.75	1.00	0.87
0.0631	**1.61**	1.19	1.71	1.39	0.72	1.44
0.0794	0.84	**0.50**	0.86	1.28	0.79	1.26
0.1000	1.25	1.05	1.61	**0.62**	1.21	0.93
0.1259	1.32	1.56	0.79	0.83	0.67	**2.08**
0.1585	0.80	1.23	1.39	1.41	1.13	1.03
0.1995	1.08	1.06	1.06	**1.61**	0.85	1.00
0.2512	1.20	0.54	0.97	0.67	0.93	0.93
0.3162	0.81	0.83	0.71	0.92	0.77	1.46
0.3982	1.07	**2.15**	0.57	1.06	0.74	1.34
0.5011	0.87	0.91	0.70	1.05	0.93	1.52
0.6309	**2.21**	1.57	**1.69**	0.95	0.54	1.17
0.7944	0.63	0.62	0.70	1.24	**0.09**	1.39
0.8913	0.74	1.11	0.86	1.14	1.23	1.52
